# High Rates of *Staphylococcus aureus* USA400 Infection, Northern Canada

**DOI:** 10.3201/eid1704.100482

**Published:** 2011-04

**Authors:** George R. Golding, Paul N. Levett, Ryan R. McDonald, James Irvine, Brian Quinn, Mandiangu Nsungu, Shirley Woods, Mohammad Khan, Marianna Ofner-Agostini, Michael R. Mulvey, the Northern Antibiotic Resistance Partnership

**Affiliations:** Author affiliations: National Microbiology Laboratory, Winnipeg, Manitoba, Canada (G.R. Golding, M.R. Mulvey);; Saskatchewan Disease Control Laboratory, Regina, Saskatchewan, Canada (P.N. Levett, R.R. McDonald);; Population Health Unit, LaRonge, Saskatchewan (J. Irvine, B. Quinn);; Northern Intertribal Health Authority, Prince Albert, Saskatchewan (M. Nsungu, S. Woods);; Kelsey Trail Health Region, Melfort, Saskatchewan (M. Khan);; Public Health Agency Canada, Ottawa, Ontario, Canada (M. Ofner-Agostini);; University of Manitoba, Winnipeg, Manitoba (M.R. Mulvey)

**Keywords:** Methicillin-resistant Staphylococcus aureus, staphylococci, MSSA, MRSA, community, Canada, NARP, bacteria, antimicrobial drug resistance, dispatch

## Abstract

Surveillance of *Staphylococcus aureus* infections in 3 northern remote communities of Saskatchewan was undertaken. Rates of methicillin-resistant infections were extremely high (146–482/10,000 population), and most (98.2%) were caused by USA400 strains. Although USA400 prevalence has diminished in the United States, this strain is continuing to predominate throughout many northern communities in Canada.

Over the past decade, community-associated methicillin-resistant *Staphylococcus aureus* (CA-MRSA) infections have rapidly emerged in Canada (*1*). These CA-MRSA strains are causing infections in often young otherwise healthy persons with no traditional health care–associated risk factors (*2*), linked with increased illness severity and deaths (*3*), and now entering and being disseminated within health care facilities (*4*). In comparison to the incidence of CA-MRSA infections in large urban centers across Canada, which has been addressed through the ongoing efforts of the Canadian Nosocomial Infection Surveillance Program (*1*), little attention has been directed at the emerging problem of CA-MRSA or CA-methicillin-susceptible *S. aureus* (MSSA) in rural and northern communities of Canada. In this study, active surveillance was undertaken in 3 remote northern communities to assess the prevalence and effects of MRSA and MSSA infections.

## The Study

**Figure 1:**
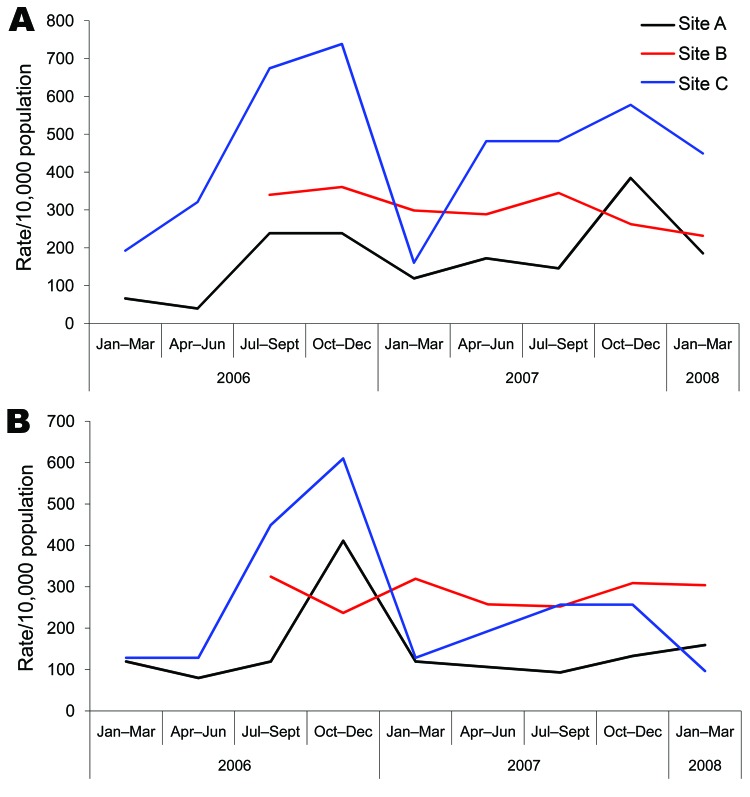
Crude rates of community-acquired methicillin-resistant *Staphylococcus aureus* (A) and methicillin-susceptible *S. aureus* (B) infections per 10,000 population in 3 select communities (sites A, B, and C) of northern Saskatchewan, Canada.

Clinically significant MRSA and MSSA isolates, identified during January 2006–March 2008, within 3 select communities (sites A–C) in northern Saskatchewan were included in this surveillance study. Site B also included 1 adjoining community, and sites A and B also included additional First Nations Reserves serviced by the community. Each site faced significant socioeconomic challenges. A total of 1,280 isolates, obtained from skin and soft tissue infections (SSTIs), urinary tract infections, upper respiratory tract infections, and lower respiratory tract infections, were identified as *S. aureus*. A high proportion of these isolates, 692 (54.1%) of 1,280, were MRSA. Over the 2-year study period, rates of MRSA and MSSA infections in the 3 communities ranged from 146–482/10,000 and 112–329/10,000 population, respectively. Trends of seasonality were apparent for MRSA infections, with the highest rates being observed in the third and fourth quarters of the year (Figure 1). Overall, the highest quarterly rates of MRSA and MSSA infections were observed at site C, with 738/10,000 and 610/10,000 population, respectively.

**Figure 2:**
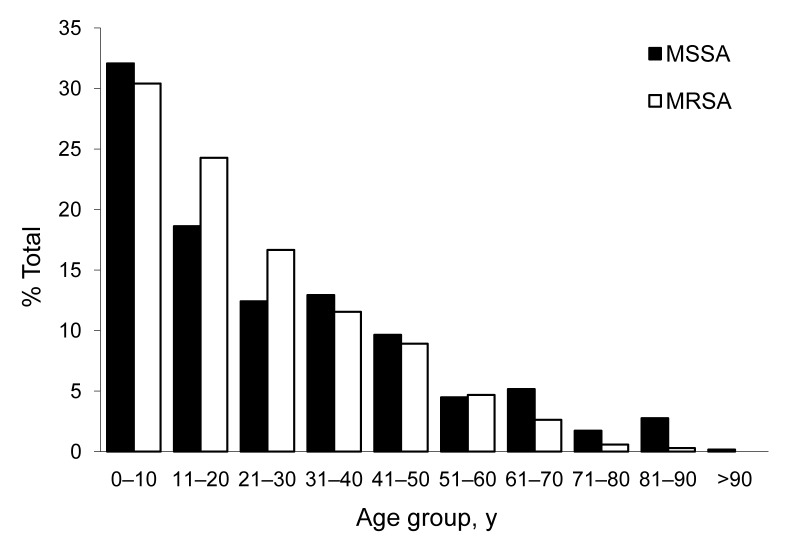
Age distribution of methicillin-resistant *Staphylococcus aureus* (MRSA) and methicillin-susceptible *S. aureus* (MSSA) infections in 3 select communities of northern Saskatchewan, Canada.

The highest proportion of MRSA (30.4%) and MSSA (32.1%) infections were identified in children <10 years of age (Figure 2). Compared to MSSA infections, MRSA infections were statistically more likely to be causing infections in persons <30 years of age (odds ratio [OR] 1.46, 95% confidence interval [CI] 1.14–1.86, p = 0.002) and less likely to be causing infections in patients >60 years of age (OR 0.33, 95% CI 0.20–0.567), p<0.001) (Figure 2). No significant difference was found in gender between those who acquired MRSA (46.7% male) and MSSA (53.3% female, 49.4% male) infections.

Most MRSA (98.6%) and MSSA (91%) isolates were obtained from SSTIs. Further analysis of SSTIs, comparing where on the body the infections were seen, showed significantly more MRSA infections in the axillae (OR 3.04, 95% CI 1.39–6.89, p = 0.004), buttocks (OR 2.1, 95% CI 1.27–3.49, p = 0.003), and trunk (OR 2.25, 95% CI 1.54–3.31, p = <0.001) than MSSA infections. MRSA infections were significantly less likely to be found in feet (OR 0.29, 95% CI 0.18–0.45, p<0.001), hands (OR 0.45, 95% CI 0.3–0.68, p<0.001), and face or head (OR 0.66, 95% CI 0.48–0.90, p = 0.009). Of the additional infection sites included in this study, MSSA infections were statistically more likely to be identified in lower respiratory tract infections (OR 5.6, 95% CI 1.5–24.62, p<0.05) and urinary tract infections (OR 6.76, 95% CI 2.87–16.71, p<0.001).

A subset of 665 isolates were further characterized by antimicrobial drug susceptibility testing (Table 1). In comparison to MSSA, MRSA were significantly more likely to be susceptible to clindamycin, erythromycin, fusidic acid, and gentamicin, but were more likely to be resistant to mupirocin (Table 1). In regards to the clindamycin-resistant isolates, 3 (18.8%) of the 16 MRSA isolates and 73 (93.6%) of the 78 MSSA isolates were inducible. For mupirocin-resistant isolates, all 328 of the MRSA isolates, but only 54 (70.1%) of the 77 MSSA isolates, displayed high level resistance (>128 μg/mL).

**Table 1:** Broth microdilution antimicrobial susceptibilities of select MRSA and MSSA isolates, northern Canada, 2006–2008*

Antimicrobial drug	MRSA isolates, n = 379					MSSA isolates, n = 286				p value	OR (95% CI)
	% R	MIC range	MIC_50_	MIC_90_		% R	MIC range	MIC_50_	MIC_90_		
Clindamycin	4.2	<0.25–>8	<0.25	<0.25		27.3	<0.25–>8	<0.25	<0.25	<0.001	0.12 (0.06–0.21)
Erythromycin	5.5	<0.25–>8	1	2		28.0	0.5–>8	2	>8	<0.001	0.15 (0.09–0.26)
Vancomycin	0	<0.25–2	1	1		0	0.5–2	1	1	NS	–
SXT	0	<0.25–2	<0.25	<0.25		0	<0.25–2	<0.25	<0.25	NS	–
Tetracycline	0.3	<2–>16	<2	<2		0	<2	<2	<2	NS	–
Ciprofloxacin	2.4	<0.12–>8	0.5	0.5		1.7	0.25–>8	0.5	0.5	NS	–
Rifampin	0	<0.25	<0.25	<0.25		0	<0.25	<0.25	<0.25	NS	–
Fusidic acid	2.1	<0.06–>8	0.12	0.25		7.7	<0.06–>8	0.25	0.5	0.001	0.26 (0.1–0.62)
Linezolid	0	0.5–4	2	4		0	1–4	4	4	NS	–
Gentamicin	1.6	<0.5–>16	<0.5	<0.5		8.0	<0.5–>16	<0.5	1	<0.001	0.18 (0.07–0.48)
Mupirocin	86.5	<0.12–>128	>128	>128		26.9	<0.12–>128	0.5	>128	<0.001	17.46 (11.56–26.43)
Synercid	0	<0.25–0.5	<0.25	0.5		0	<0.25–1	0.5	0.5	NS	–
Nitrofurantoin	0	<32	<32	<32		0	<32	<32	<32	NS	–

*Values are mg/mL except as indicated. MRSA, methicilllin-resistant *Staphylococcus aureus*; MSSA, methicillin-susceptible *S. aureus*; R, resistant; OR, odds ratio; CI, confidence interval; MIC_50_, 50% minimum inhibitory concentration; MIC_90_, 90% inhibitory concentration; SXT, sulfamethoxazole/trimethoprim; NS, not significant.

Pulsed-field gel electrophoresis (PFGE) showed that most MRSA isolates (372/379, 98.2%) were USA400. The remaining 7 MRSA isolates were identified as CMRSA10 (USA300, sequence type (ST) 8) (n = 5), CMRSA2 (USA100/800, ST5) (n = 1), and CMRSA8 (EMRSA15, ST22) (n = 1). As anticipated, PFGE revealed much greater genetic diversity among the MSSA strains circulating in these regions than in MRSA strains. Notably, however, most MSSA PFGE fingerprints (79.2%) were related to highly successful Canadian epidemic MRSA strains, a finding that was further confirmed by using *spa* typing (*5*) (Table 2).

**Table 2:** Relationship of molecularly characterized MSSA isolates to MRSA epidemic strain types*

MRSA PFGE epidemic types (MLST)	No. (%) related MSSA isolates	PVL positive	Predominant *spa* type†
CMRSA1/USA600 (ST45)	38 (13.3)	0	t065 (n = 23)
CMRSA2/USA100/800 (ST5)	77 (26.9)	0	t311 (n = 46)
CMRSA4/USA200 (ST36)	30 (10.5)	0	t012 (n = 12)
CMRSA7/USA400 (ST1)	12 (4.2)	12	t128 (n = 8)
CMRSA10/USA300 (ST8)	3 (1.1)	2	t008 (n = 2)
USA700 (ST72)	1(0.4)	0	t148 (n = 1)
ST97	18 (6.3)	0	t2728 (n = 11)
USA1000 (ST59)	33 (11.5)	1	t163 (n = 27)
USA1100 (ST30)	1 (0.4)	0	t122 (n = 1)

*n = 286 MSSA isolates. MSSA, methicilin-susceptible *Staphylococcus aureus*; MRSA, methicillin-resistant *S. aureus*; PVL, Panton-Valentine leukocidin; PFGE, pulsed-field gel electrophoresis; MLST, multilocus sequence typing; ST, sequence type. †www.ridom.de.

MRSA isolates were more likely to harbor the genes encoding Panton-Valentine leukocidin than were MSSA isolates, 95.5% versus 5.2%, respectively. The PFGE and *spa* types of the 15 Panton-Valentine leukocidin–positive MSSA isolates were associated with the CA-MRSA epidemic strain types USA400, USA300, and USA1000 (Table 2).

## Conclusions

Rates of MSSA and MRSA infections in these 3 northern Saskatchewan communities (112–482 cases/10,000 population) far exceed MRSA rates reported in the neighboring provinces of Manitoba (≈16/10,000 population) (*6*) and Alberta (10.7/10,000 population) (*7*), as well as benchmark hospital rates provided by the Canadian Nosocomial Infection Surveillance Program (3.43 cases/10,000 patient days) (*1*). The high rates of *S. aureus* infections in remote northern Saskatchewan communities has been attributed to a combination of risk factors, including overcrowding and poor housing conditions, inadequate hygiene, preexisting skin conditions, and previous high usage of antimicrobial drugs (*8*).

USA400 was by far the predominant strain type in all 3 communities, accounting for >98% of the MRSA isolates. USA400 was first reported in Manitoba as an outbreak in the southern region in the late 1990s, but has since spread to the northern regions of the province from 2000 to 2004 (*9*). USA400 was thereafter seen in a central eastern Saskatchewan community adjacent to the Manitoba border (*2*) and has since disseminated as far north as Nunavut (*10*) and southwestern Alaska (*11*).

Because MRSA and MSSA SSTIs tended to be identified more frequently from different body sites, it is appealing to speculate that CA-MRSA strains, such as USA400, might also colonize different body sites (e.g., axillae or intestines) more efficiently than other strains of *S. aureus*. This hypothesis coincides with a recent report in which nasal colonization was less likely in patients with CA-MRSA SSTIs than in those with hospital-acquired MRSA SSTIs (*12*). Intestinal carriage of *S. aureus* has been implicated as a risk factor for infection (*13*) and could be a strong contributor to environmental dissemination and transmission (*14*). This possibility was recently further supported by the results of a study in which the rectal carriage, but not nasal carriage, of USA300 was strongly associated with SSTIs in children (*15*). Further study is required to determine whether specific lineages of *S. aureus* are more proficient colonizers at non-nasal colonization sites, what host/bacteria genetic factors are involved, and whether this colonization plays a role in the high success of these CA-MRSA strain types.

To address the high rates of *S. aureus* infections in northern Saskatchewan, physician treatment algorithms and educational materials have been provided throughout many northern communities and schools in Saskatchewan. These materials are all freely available (www.narp.ca) and are intended to promote proper antimicrobial drug usage and hygiene to diminish the spread of *S. aureus* disease.
